# Supplementary material for: Milk and Meat Allergens from Bos taurus β-Lactoglobulin, α-Casein, and Bovine Serum Albumin: An In-Vivo Study of the Immune Response in Mice
Source: Nutrients. 2019 Sep 4;11(9):2095. doi: 10.3390/nu11092095 (PMC6769769; doi:10.3390/nu11092095)
Supplement: Supplementary file 1 [file nutrients-11-02095-s001.pdf]

nutrients

Supplementary Materials

Table S1. Primers and PCR conditions.

Target	Primer	Sequence (5′ → 3′)	T_a [°C]	$MgCl_2$ [mM]	Reference
Total bacteria	UniF	gtgcgcayggyygtcgtca	60	2.0	a
	UniR	acgtcrtccmcnccttcctc			
Clostridium leptum group	sg-Clept-F	gcacaagcagtggagt	53	2.0	b
	sg-Clept-R3	cttcctccgttttgtcaa			
Bacteroides–Prevotella–Porphyromonas group	BPP-F	ggtgtcggcttaagtgccat	56	3.0	c
	BPP-R	cgga(c/t)gtaagggccgtgc			
Clostridium coccoides group	g-ccoc-F	aaatgacggtacctgactaa	58	5.0	c
	g-ccoc-R	ctttgagtttcattcttgcgaa			
Bifidobacterium	BIF-F	tcg cgtc(c/t)ggtgtgaaag	58	2.0	c
	BIF-R	ccacatccagc(a/g)tccac			
Lactobacillus	Lac1F	agcagtagggaatcttcca	58	2.0	d
	Lab667	caccgctacacatggag			

a. Fuller, Z., Louis, P., Mihajlovski, A., Rungapamestry, V., Ratcliffe, B., Duncan, A. J. (2007). Influence of cabbage processing methods and prebiotic manipulation of colonic microflora on glucosinolate breakdown in man. *British Journal of Nutrition*. *98(2)*, 364-72.

b. Shen, J., Zhang, B., Wei, G., Pang, X., Wei, H., Li, M., Zhang, Z., Jia, W., Zhao, L. (2006). Molecular profiling of the *Clostridium leptum* subgroup in human fecal microflora by PCR-Denaturing Gradient Gel Electrophoresis and clone library analysis. *Applied and Environmental Microbiology*. *72(8)*, 5232–5238.

c. Rinttila, T., Kassinen, A., Malinen, E., Krogius, L., Palva, A. (2004). Development of an extensive set of 16S rDNA-targeted primers for quantification of pathogenic and indigenous bacteria in faecal samples by real-time PCR. *Journal of Applied Microbiology*. *97*, 1166–1177.

d. Heilig, H. G. J., Zoetendal, E. G., Vaughan, E. E., Marteau, P., Akkermans, A. D. L., de Vos, W. M. (2002). Molecular diversity of *Lactobacillus* spp. and other lactic acid bacteria in the human intestine as determined by specific amplification of 16S ribosomal DNA. *Applied and Environmental Microbiology*. *68(1)*, 114–123.
